# Laparoscopic Drainage of a Hepatic Echinococcal Cyst: A Case Report

**DOI:** 10.1155/2011/107087

**Published:** 2011-07-28

**Authors:** Steven B. Goldin, James J. L. Mateka, Michael J. Schnaus, Sujat Dahal

**Affiliations:** Department of Surgery, College of Medicine, University of South Florida, Tampa, FL 33606, USA

## Abstract

The Echinococcus granulosus tapeworm causes hepatic echinococcosis. It is endemic in the Mediterranean region, Middle East, and South America. Human infection is secondary to accidental consumption of ova in feces. Absorption through the bowel wall and entrance into the portal circulation leads to liver infection. This case involves a 34 y/o Moroccan male with an echinococcal liver cyst. His chief complaint was RUQ pain. The patient was treated with albendazole and praziquantel. His PMH and PSH was noncontributory. Patient was not on any other medications. ROS was otherwise unremarkable. The patient was AF VSS. He was tender to palpation in RUQ. Liver function tests were normal. Echinococcal titers were positive. CT demonstrated a large cystic lesion in the right lobe of the liver measuring 13.5 cm in diameter. The patient underwent successful laparoscopic drainage and excision of echinococcal cyst. Final pathology demonstrated degenerating parasites (E. granulosus) of echinococcal cyst.

## 1. Introduction

Hepatic echinococcus is acquired by humans secondary to accidental consumption of ova in dog feces [1]. It results from infection by the dog tapeworm. It is endemic in sheep and cattle raising areas in Europe, China, Russia, the Mediterranean region, Middle East, South America, Australia, New Zealand, and Southern Africa. It is exceedingly rare in the United States [2] and mainly occurs in members of high risk groups such as sheep farmers in the west, some native American Indians in the southwest and Alaska, and in immigrants [3]. Its incidence is estimated to be less than 1 case per 1 million population in the continental United States and can range from 1 to 220 cases per 100,000 persons in endemic areas. Hepatic echinococcal cysts are described most frequently using the ultrasonographic classification system of Gharbi et al. [4] (Table 1). Based on the Gharbi classification system, the World Health Organization (WHO) Informal Working Group on Echinococcosis modified their International Classification System [5] to identify the functional state of parasites to define treatment (Table 2).

## 2. Case Report

**Figure 1:**
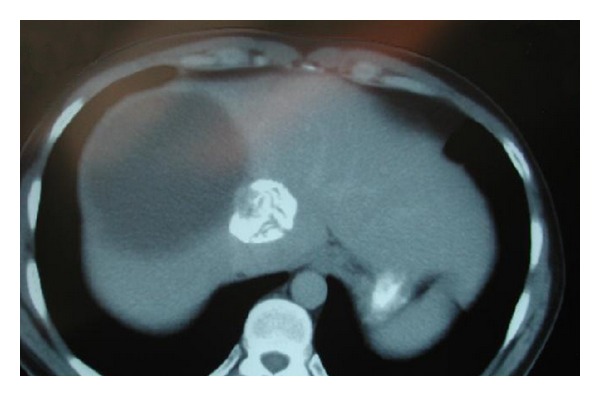
CT after 3 month course of albendazole and praziquantel.

A 34-year-old Moroccan male presented with a several month history of moderately severe, constant, and progressively worsening, dull aching right upper quadrant pain (RUQ). There were no alleviating and precipitating factors. A computed tomography (CT) scan revealed a 13.5 cm cystic lesion in the right lobe of the liver (Gharbi Type I) and densely calcified masses in the right and left sides of the liver (Gharbi Type V). An ELISA test for echinococcal infection was positive. The patient was treated with albendazole and praziquantel for 3 months with no clinical or radiographic improvement (Figure 1). Laparoscopic cyst evacuation and partial cyst excision was undertaken.

**Figure 2:**
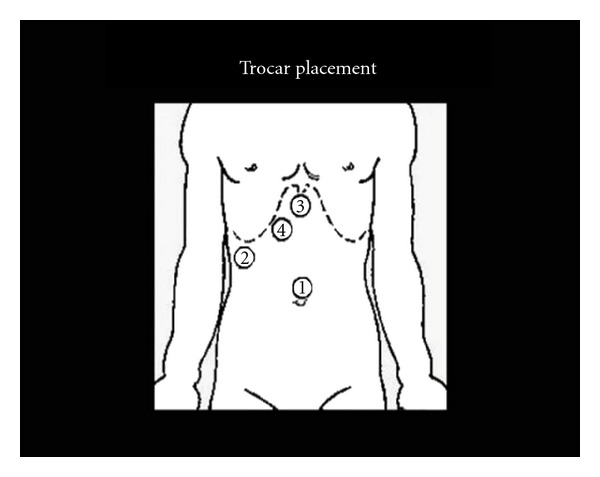
Trocar placement.

**Figure 3:**
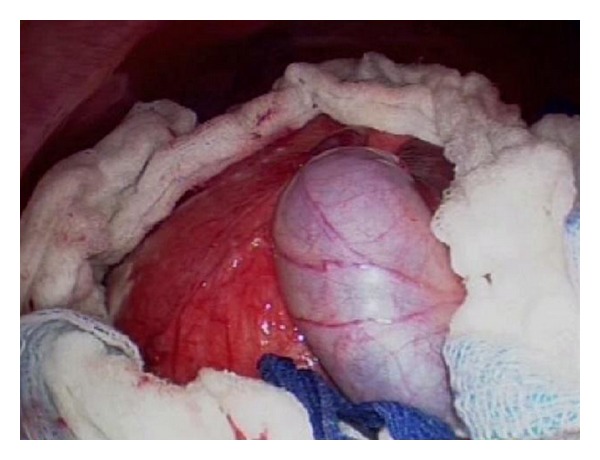
Cyst surrounded by hypertonic saline-soaked pediatric laparotomy pads.

**Figure 4:**
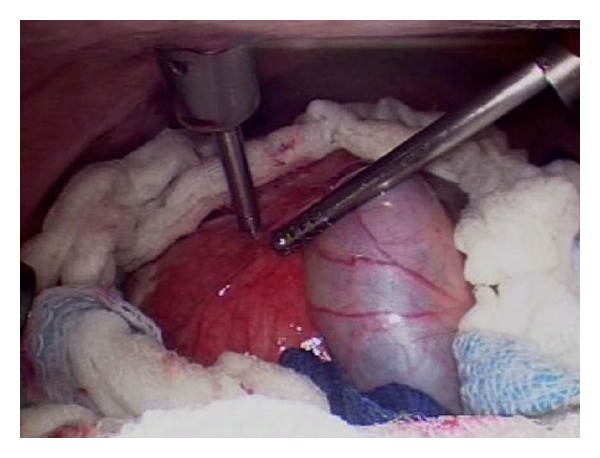
Aspiration of cyst contents and instillation of hypertonic saline.

**Figure 5:**
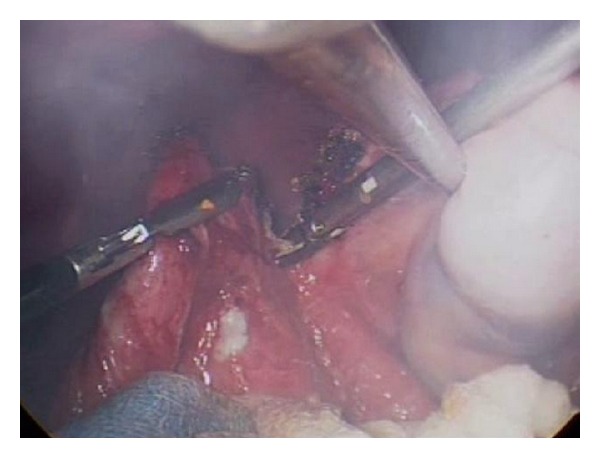
Excision of cyst wall using a harmonic scalpel.

**Figure 6:**
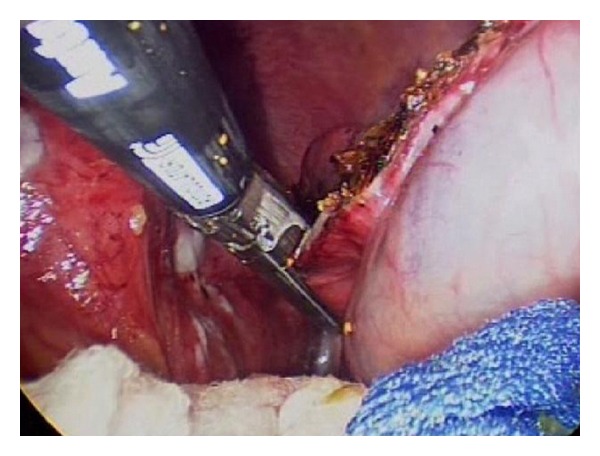
Excision of cyst wall using endo-GIA stapler with 2.5 mm vascular loads.

The procedure was done using four 12 mm ports as shown in Figure 2. The cyst was first surrounded with pads soaked in a scolicidal agent (20% hypertonic saline) (Figure 3), to protect the abdominal contents from contamination. Approximately, 400 mL was aspirated from the cyst, which was then filled with 1 liter of 20% hypertonic saline (Figure 4). This saline was aspirated, and the cyst refilled again with hypertonic saline. The saline was allowed to dwell in the cyst cavity for 30 minutes per cycle to ensure complete killing of organisms. The cyst was then opened and a portion of the cyst wall excised using a harmonic scalpel and endo-GIA stapler with 2.5 mm vascular loads (Figures 5 and 6). A “double bag” technique was used to remove the excised cyst wall and debris, which were placed into an Endo CATCH bag and a Lapsac Surgical Tissue Pouch prior to being removed from the abdomen. An argon beam coagulator was then used on the entire remaining cyst wall. A Yankauer sucker was placed through the midepigastric port site, to aid in irrigation of the abdomen with a Clorpactin solution. A 10 French flat Jackson-Pratt drain was placed in the cyst bed. 

The patient was discharged on postoperative day three with the drain, which had collected a small amount of bilious fluid. The drain was removed after seven days. Pathology showed degenerating stage cysts without any living forms of E. granulosus. The infectious disease service recommended 28 more days of albendazole. At six-month followup, the patient remained asymptomatic and disease-free.

## 3. Discussion

Surgery is the primary treatment for echinococcal disease. However, controversy still surrounds the preoperative medical treatment and type of operative procedure. The role of preoperative endoscopic retrograde cholangiopancreatography (ERCP) continues to be debated, with proposed benefits including preoperative definition of cystobiliary relationships, treatment of cholangitis and biliary obstruction, and possible definitive treatment in cases of intrabiliary rupture. In one study, ERCP was reported to be safe method to treat biliary complications of hepatic hydatidosis before and after surgical management [6]. ERCP, however, may cause complications and has a false negative rate of 17%–20% in identifying small cystobiliary communications due to elevated cyst pressure, minimal communication, or transient obstruction of the communication by daughter cysts [7]. Therefore, even if no cystobiliary communications are identified preoperatively, cysts must still be carefully inspected intraoperatively, since small cystobiliary communications develop in 80%–90% of patients with hepatic echinococcal disease. 

Biliary obstruction only occurs in 5%–10% of patients due to biliary communications that are at least 5 mm in diameter. Communications of this size or larger are more significant due to their ability to carry hydatid debris and daughter cysts into the common bile duct causing biliary obstruction in over 66.6% of cases. In patients without jaundice or cholangitis, ERCP findings are normal in 50% and demonstrate cystobiliary rupture, biloma, or bile duct compression in the other 50% [8, 9]. Özaslan and Bayraktar [7] suggest that preoperative or postoperative ERCP should be used only for complicated cases and that for uncomplicated cases, routine use of ERCP should not be recommended except for surgery planning. Another recent study suggested that cyst diameter independently predicts risk for biliary-cyst communication in asymptomatic patients. The mean cyst size in patients with biliary leakage was 10.2 cm, compared to 6.1 cm in patients without biliary leakage, and they suggested that preoperative ERCP should only be used in asymptomatic patients large cysts [10]. 

Use of an intraoperative cholangiogram is also controversial. Ramachandran and Arora suggest that an intraoperative cholangiogram is unnecessary and increases morbidity [11]. They state that inspection of the cyst wall using the laparoscope will identify all major bile leaks. Ozmen and Coskun [12] also states biliary tract communications can be controlled by inspection of the cyst cavity and ligation of ruptured bile ducts less than 5 mm in diameter. If bile ducts greater than 5 mm in diameter are identified, intraoperative cholangiogram is done to assess the common bile duct (CBD) for debris, which requires CBD exploration with T-tube placement if present. 5 mm is used as a cutoff as bile ducts smaller than this rarely transmit particulate matter to the CBD, while 65% of bile ducts 5 mm or larger allow passage of material into the CBD. 

All surgical treatments require complete cyst exposure, cyst decompression evacuation and sterilization, ligation of bile duct communications, and cavity management [13]. Open procedures can be classified into (1) conservative tissue sparing techniques that remove the parasite and leave the majority of the pericyst in place and (2) radical procedures that extricate the entire pericyst. Conservative techniques include partial cystopericystectomy and near-total pericystectomy. Radical procedures such as cystopericystectomy, hepatic lobectomy, and hepatectomy have been used in the past. Currently, they are rarely used and are being replaced by cystotomy, partial cystectomy, and omentoplasty, which can all be done laparoscopically. Advantages of the laparoscopic procedures include less pain, good cosmetic results, rapid recovery, and decreased complications.

The chosen operative procedure depends on the location, size, type of cyst, and the surgeon's skills [14]. Total pericystectomy is often avoided if the pericystic area is near major vascular or biliary structures because of a high risk of severe bleeding and bile duct injury. A total pericystectomy, however, is considered by some to be preferable due to its low risk of recurrence, lower risk of biliary leakage, reduced inflammatory complications, and increased rate of detection of daughter cysts [15–17]. The higher risk of total pericystectomy limits some surgeons to recommend this only for small peritoneal cysts or cyst on the exterior surface of the liver [18]. Another radical surgical procedure is hepatic resection, but it is accompanied by a high morbidity rate [13]. Liver resection is suggested when a complete lobe is involved or when other procedures have failed [18]. Alonso Casado et al. suggested radical resection for hepatic hydatid cyst has better outcomes than puncture-aspiration-injection-re-aspiration (PAIR) or partial resection regarding morbidity and mortality with almost no recurrence rate [19]. 

 Laparoscopic treatments that have been described include cystotomy, partial pericystectomy, and total pericystectomy [11, 13]. Laparoscopic techniques are gaining popularity even though no fail-safe methodology has been devised to completely ensure the prevention of cyst spillage. A laparoscopic hand-assisted procedure has been suggested to prevent intra-abdominal spillage [20]. Others suggest using antiscolecoidal agents preoperatively and intraoperatively to completely eradicate the parasite, but this may cause sclerosing cholangitis [21, 22]. In all procedures, it is the initial penetration and aspiration of the cyst fluid that remains the most difficult part of the procedure [22]. Some suggest using a special umbrella trocar to secure the cyst to the abdominal wall along with a special suction device. Another method uses an antiscolecoidal fluid (Cetrimide) and Trendelenburg positioning, but has failed to prevent stray jets of fluid from escaping [23]. The only cysts not removed laparoscopically are deep intraparenchymal cysts close to the vena cava, or cysts containing thick, calcified walls [24, 25]. The decision is often made, to perform partial pericystectomy and not to treat thickly calcified cysts in close approximation to major vascular and biliary structures due to the high risk of severe complications [13, 26]. 

Good laparoscopic candidates include those with superficial and fluid filled cysts, while deep cysts should be approached in an open manner due to the risk of hemorrhage [27]. Additional exclusion criteria for laparoscopic intervention include the presence of more than three cysts, and cysts with thick and/or calcified walls [13]. In 2000, Seven et al. established that laparoscopy could be used to treat hepatic echinococcal cysts with morbidity and recurrence rates comparable to those observed in open series [28]. In 2002, Kayaalp demonstrated that successful cyst evacuation could be accomplished at a rate of 92% in anteriorly located cysts and a rate of 73% in posterior-superiorly located cysts [29]. A series of 18 patients treated with laparoscopic surgical techniques similar to the ones instituted with our patient demonstrated safe results with low rates of conversion to open techniques [30]. A study out of Amsterdam demonstrated that laparoscopic treatment of anteriorly located hepatic cysts had a success rate of 77%–100%, with low complication and recurrence rates (0%–17% and 1%–9%, resp.) [31]. However, lower complication and recurrence rates of the laparoscopic approach compared to open procedures can be misleading due to bias in patient selection criteria [32].

Lastly, percutaneous methods are becoming increasingly popular. The procedure, initially developed by Ben Amor et al. is called PAIR: puncture-aspiration-injection-re-aspiration [33]. The patient selection is crucial to its success. The increased popularity of percutaneous aspiration is due to the advances in imaging techniques such as CT and ultrasound, development of fine needles and catheters, and the origination of the intercostal intrahepatic approach which have decreased the risk of peritoneal spillage and anaphylaxis [34]. 

Percutaneous drainage of hydatid cysts involves aspiration of the cyst fluid and injection of contrast to ensure no large biliary tract communication. The cyst is then infused with an antiscolecoidal agent and drained with a catheter [13, 23]. Only type I and type II cysts and some of type III and type IV cysts can be managed using PAIR. In high surgical risk patients such as pregnant women and those with several cysts, percutaneous drainage might prove advantageous [13]. Benefits of PAIR include a low recurrence rate, low morbidity rate, short hospitalization, and less scarring [23, 35, 36]. Some argue that the percutaneous methods produces equivalent results to laparoscopic surgery and that surgery should be reserved for situations when percutaneous treatment is not available or has failed [31]. Giorgio et al. presented a modified technique with a better overall outcome called double puncture-aspiration-injection (D-PAI) [37]. Fine-needle puncture of the hepatic liver cyst is repeated 3 days after the initial aspiration, and the second half of the procedure does not include reaspiration of the scolicidal agent (95% sterile alcohol) or catheter drainage [38].

## 4. Summary

Optimal treatment algorithms for hepatic echinococcal cystic disease are not fully defined. Controversies revolve around the use of ERCP and open surgical, laparoscopic, or percutaneous procedures. Although the efficacy of specific laparoscopic techniques remains a debated topic, it has been demonstrated at multiple centers around the world that the principles of open surgical treatment of hepatic echinococcus can be adhered to by laparoscopic intervention.

## Figures and Tables

**Table 1:** Gharbi classification of hepatic echinococcal cysts.

Type I	Pure fluid
Type II	Fluid collection, spilt-wall floating membrane
Type III	Fluid collection with septa, daughter cysts, and honeycomb image
Type IV	Heterogeneous echographic pattern
Type V	Reflecting thick walls

**Table 2:** International classification of ultrasound images in cystic echinococcosis for application in clinical and field epidemiological settings.

Tybe of Cyst	Status	Ultrasound features	Remarks
CL	Active	Signs no pathognomonic, unilocular, and no cyst wall	Usually early stage, not fertile, and differential diagnosis necessary
CE 1	Active	Cyst wall, hydatid sand	Usually fertile
CE 2	Active	Multivesicular, c st wall, and “rosette-like”	Usually fertile
CE 3	Transitional	Detachment of laminated membrane, “water-lil sign,” less round-decreased intracystic pressure	Starting to degenerate, may produce daughter cysts
CE 4	Inactive	Heterogeneous hypo- or hyperechogenic degenerative contents, no daughter cysts	Usually no living protoscoleces, differential diagnosis necessary
CE 5	Inactive	Thick calcified wall, calcification partial to complete, and not pathognomonic but highly suggestive of diagnosis	Usually no living protoscoleces

## References

[1] Bouree P (2001). Hydatidosis: dynamics of transmission. *World Journal of Surgery*.

[2] Trolan W (2009). R. Echinococcosis. *eMedicine*.

[3] Chrieki M (2002). Echinococcosis—an emerging parasite in the immigrant population. *American Family Physician*.

[4] Gharbi HA, Hassine W, Brauner MW, Dupuch K (1981). Ultrasound examination of the hydatic liver. *Radiology*.

[5] MacPherson CNL, Vuitton DA, Gharbi HA (2003). International classification of ultrasound images in cystic echinococcosis for application in clinical and field epidemiological settings. *Acta Tropica*.

[6] Goumas K, Poulou A, Dandakis D (2007). Role of endoscopic intervention in biliary complications of hepatic hydatid cyst disease. *Scandinavian Journal of Gastroenterology*.

[7] Özaslan E, Bayraktar Y (2002). Endoscopic therapy in the management of hepatobiliary hydatid disease. *Journal of Clinical Gastroenterology*.

[8] Ponchon T, Bory R, Chavaillon A (1987). Endoscopic retrograde cholangiography and sphincterotomy for complicated hepatic hydatid cyst. *Endoscopy*.

[9] Uccheddu A, Murgia C, Licheri S, Dazzi C, Cagetti M (1989). Endoscopic retrograde cholangiography in the diagnosis of the liver hydatidosis. *Giornale di Chirurgia*.

[10] Kilic M, Yoldas O, Koc M (2008). Can biliary-cyst communication be predicted before surgery for hepatic hydatid disease: does size matter?. *American Journal of Surgery*.

[11] Ramachandran CS, Arora V (2001). Laparoscopic surgery in hepatic hydatid cysts: a technical improvement. *Surgical Laparoscopy, Endoscopy and Percutaneous Techniques*.

[12] Ozmen MM, Coskun F (2002). New technique for finding the ruptured bile duct into the liver cysts: scope in the cave technique. *Surgical Laparoscopy, Endoscopy and Percutaneous Techniques*.

[13] Dervenis C, Delis S, Avgerinos C, Madariaga J, Milicevic M (2005). Changing concepts in the management of liver hydatid disease. *Journal of Gastrointestinal Surgery*.

[14] Meyers WC, kim RD, Townsend CM0 (2001). Echinococcal cyst. *Sabiston Textbook of Surgery: The Biological Basis of Modern Surgical Practice*.

[15] Cirenei A, Bertoldi I (2001). Evolution of surgery for liver hydatidosis from 1950 to today: analysis of a personal experience. *World Journal of Surgery*.

[16] Dervenis C, Delis S, Avgerinos C, Madariaga J, Milicevic M (2005). Changing concepts in the management of liver hydatid disease. *Journal of Gastrointestinal Surgery*.

[17] Prousalidis J, Tzardinoglou E, Kosmidis C, Katsohis K, Aletras O (1999). Surgical management of calcified hydatid cysts of the liver. *HPB Surgery*.

[18] Safioleas M, Misiakos E, Manti C, Katsikas D, Skalkeas G, Moreno-Gonzalez E (1994). Diagnostic evaluation and surgical management of hydatid disease of the liver. *World Journal of Surgery*.

[19] Alonso Casado O, Moreno González E, Loinaz Segurola C (2001). Results of 22 years of experience in radical surgical treatment of hepatic hydatid cysts. *Hepato-Gastroenterology*.

[20] Bensaadi H, Champault G (2004). Laparoscopic Hand-Assisted Surgery for Hydatid Cysts of the Liver. *Surgical Laparoscopy, Endoscopy and Percutaneous Techniques*.

[21] Aktan AÖ, Yalin R (1996). Preoperative albendazole treatment for liver hydatid disease decreases the viability of the cyst. *European Journal of Gastroenterology and Hepatology*.

[22] Sağlam A (1996). Laparoscopic treatment of liver hydatid cysts. *Surgical Laparoscopy, Endoscopy and Percutaneous Techniques*.

[23] Bickel A, Loberant N, Lujan-Mompean JA (1994). Laparoscopic treatment of a liver hydatid cyst. *British Journal of Surgery*.

[24] Ertem M, Uras C, Karahasanoglu T, Erguney S, Alemdaroglu K (1998). Laparoscopic approach to hepatic hydatid disease. *Digestive Surgery*.

[25] Lujan Mompean JA, Parrilla Paricio P, Robles Campos R, Garcia Ayllon J (1993). Laparoscopic treatment of a liver hydated cyst. *British Journal of Surgery*.

[26] Uravic M, Stimac D, Lenac T (1998). Diagnosis and treatment of liver hydatid disease. *Hepato-Gastroenterology*.

[27] Sayek I, Cakmakci M (1999). Laparoscopic treatment of echinococcal cysts of the liver. *Zentralblatt fur Chirurgie*.

[28] Seven R, Berber E, Mercan S, Eminoglu L, Budak D (2000). Laparoscopic treatment of hepatic hydatid cysts. *Surgery*.

[29] Kayaalp C (2002). Evacuation of hydatid liver cysts using laparoscopic trocar. *World Journal of Surgery*.

[30] Baskaran V, Patnaik PK (2004). Feasibility and safety of laparoscopic management of hydatid disease of the liver. *Journal of the Society of Laparoendoscopic Surgeons*.

[31] Schipper HG, Kager PA (2004). Diagnosis and treatment of hepatic echinococcosis: an overview. *Scandinavian Journal of Gastroenterology, Supplement*.

[32] Acarli K (2004). Controversies in the laparoscopic treatment of hepatic hydatid disease. *HPB*.

[33] Ben Amor N, Gargouri M, Gharbi HA, Golvan YJ, Ayachi K, Kchouck H (1986). Trial therapy of inoperable abdominal hydatid cysts by puncture. *Annales de Parasitologie Humaine et Comparee*.

[34] Men S, Hekimoğlu B, Yücesoy C, Arda IS, Baran I (1999). Percutaneous treatment of hepatic hydatid cysts: an alternative to surgery. *American Journal of Roentgenology*.

[35] Khuroo MS, Zargar SA, Mahajan R (1991). Echinococcus granulosus cysts in the liver: management with percutaneous drainage. *Radiology*.

[36] Wang X, Li Y, Feng S (1994). Clinical treatment of hepatic and abdominal hydatid cyst by percutaneous puncture, drainage and curettage. *Chinese Journal of Parasitology & Parasitic Diseases*.

[37] Giorgio A, Tarantino L, Francica G (1992). Unilocular hydatid liver cysts: treatment with US-guided, double percutaneous aspiration and alcohol injection. *Radiology*.

[38] Giorgio A, De Stefano G, Esposito V (2008). Long-term results of percutaneous treatment of hydatid liver cysts: a single center 17 years experience. *Infection*.

